# Topical minocycline foam: A new option for acne treatment?

**DOI:** 10.1111/jdv.20637

**Published:** 2025-04-25

**Authors:** Evgenia Makrantonaki

**Affiliations:** ^1^ Derma Center Wildeshausen Wildeshausen Germany; ^2^ Departments of Dermatology, Venereology, Allergology and Immunology, Staedtisches Klinikum Dessau Brandenburg Medical School Theodor Fontane and Faculty of Health Sciences Brandenburg Dessau Germany; ^3^ Department of Dermatology and Allergology Ulm University Ulm Germany

Acne vulgaris (AV) is a chronic inflammatory disorder of the pilosebaceous unit and is one of the most common dermatological conditions worldwide, affecting an estimated 650 million people.^1^, ^2^ Abnormalities in several processes (inflammation, sebum production, sebocyte differentiation and *P. acnes* proliferation) may contribute to the development of acne; hence, most patients require a multi‐pronged treatment regimen.^1^, ^2^ However, the complexity of these regimens can often lead to non‐adherence, which is a key factor in treatment failure. Acne is known to lead to scarring and post‐inflammatory hyperpigmentation, which can subsequently impact a patient's quality of life. It is therefore vital that treatment is initiated early. While systemic antibiotics, particularly tetracyclines, have historically been a cornerstone of treatment for moderate‐to‐severe AV, concerns regarding antibiotic resistance and systemic adverse effects (e.g. gastrointestinal, nervous system and liver toxicity) underscore the need for alternative treatment strategies.

In this context, the study by Le et al.^3^ evaluating the efficacy and safety of topical minocycline foam (FMX101 4%) in Chinese patients with moderate‐to‐severe facial AV offers a promising therapeutic approach. This phase 3, multi‐centre, randomized, double‐blind, vehicle‐controlled study demonstrated that FMX101 4% significantly reduced the inflammatory lesion count (ILC) at Week 12 compared with vehicle foam (ILC = −21.0 vs. −12.3, *p* < 0.001). In addition, the Investigator's Global Assessment (IGA) success rate was significantly higher in the treatment group (8.06% vs. 0%, *p* = 0.002), supporting the clinical efficacy of the formulation. The study also confirmed a favourable safety profile, with treatment‐emergent adverse events (TEAEs) predominantly mild to moderate and comparable between the two groups. No treatment‐emergent serious adverse events were reported, further demonstrating FMX101 4% as a well‐tolerated option.

The present findings are consistent with those of prior studies conducted outside China, wherein FMX101 4% demonstrated significant efficacy and safety benefits.^4^, ^5^ However, variations in IGA success rates across studies underscore the necessity for standardized assessment protocols to minimize subjective bias. A notable advantage of FMX101 4% is its capacity to deliver minocycline directly to the pilosebaceous unit while minimizing systemic absorption, thereby reducing the risk of antibiotic resistance and systemic toxicity. This characteristic is of particular relevance in the context of long‐term AV management, where the sustained efficacy and tolerability of the treatment are of paramount importance. The findings of the study support the integration of FMX101 4% into clinical practice as an effective and safer alternative to systemic tetracyclines, especially for patients with contraindications to oral antibiotics. Nevertheless, some limitations warrant consideration. The study's follow‐up period was limited to 12 weeks, leaving long‐term efficacy and relapse rates unaddressed. Additionally, while the study population was well characterized, further research should explore FMX101 4% in broader patient demographics, including adolescents and those with varying AV severities.

In conclusion, the study by Le et al. provides compelling evidence to support the hypothesis that FMX101 4% is a viable topical therapy for moderate‐to‐severe AV in Chinese patients. Given its favourable safety profile and significant clinical efficacy, FMX101 4% represents a significant advancement in dermatological therapeutics, offering a targeted, well‐tolerated alternative to traditional systemic treatments.

## FUNDING INFORMATION

None to declare.

## CONFLICT OF INTEREST STATEMENT

None to declare.

## Data Availability

Data sharing is not applicable to this article as no new data were created or analysed in this study.
